# Re‐Innovation in Clinical Trial Designs Based on Precision Therapy

**DOI:** 10.1002/cai2.70028

**Published:** 2025-09-24

**Authors:** Shenglan Li, Jiachen Wang, Zhuang Kang, Xun Kang, Feng Chen, Wenbin Li

**Affiliations:** ^1^ Department of Neuro‐Oncology, Cancer Center, Beijing Tiantan Hospital Capital Medical University Beijing China

**Keywords:** adaptive trials, biomarkers, clinical trials, precision medicine

## Abstract

This article, centered on precision therapy, argues that traditional disease‐centered clinical trials are flawed with prolonged cycles, insufficient early‐phase samples, and inflexible protocols, thus proposing the concept of “clinical trial re‐innovation.” It elaborates that this re‐innovation, driven by biomarker technology, multi‐arm multi‐stage designs, and improved trial flexibility, is realized through innovative designs like basket, umbrella, and platform trials, and explores their application in multiple diseases with cases, aiming to advance precise and efficient clinical research and improve patient outcomes.

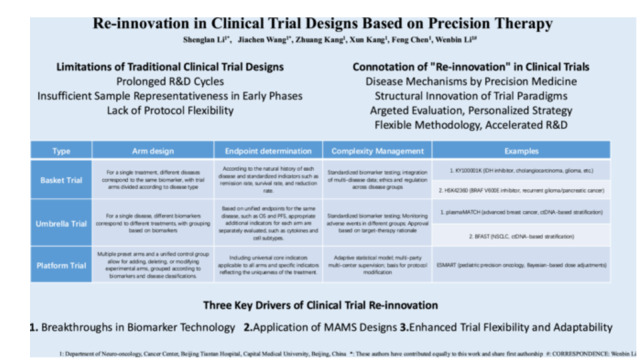

**Abbreviations**
AMPK: adenosine monophosphate‐activated protein kinase
BFAST: Blood First Assay Screening Trial
cf‐tDNA: cell‐free tumor DNA
CHA: chlorogenic acid
CSF: cerebrospinal fluid
ctDNA: circulating tumor DNA
ESMART: European Proof‐of‐concept Therapeutic Stratification Trial of Molecular Anomalies in Relapsed or Refractory Tumors
FDA: Food and Drug Administration
H3K27M: Histone H3.3K27M
ICH: International Council for Harmonisation of Technical Requirements for Pharmaceuticals for Human Use
LAR: luminal androgen receptor
MAMS: multi‐arm multi‐stage
NFκB: nuclear factor kappa‐B
Nrf2: nuclear factor erythroid 2‐related factor 2
NSCLC: non‐small cell lung cancer
OS: overall survival
PFS: progression‐free survival
TNBC: triple‐negative breast cancer
VAF: variant allele fraction

## 1 Introduction

### 1.1 Background for the Transformation of Clinical Trial Design in the Era of Precision Medicine

In the realm of precision therapy, the determination of optimal treatment strategies is increasingly predicated on individualized patient characteristics, including genetic mutations and biomarkers. Traditional clinical trial designs, typically adhering to a disease‐centric model with standardized methodologies, are undergoing reevaluation due to the progress in multi‐omics sequencing technologies. These technological breakthroughs have disclosed that remarkable molecular heterogeneity persists within the same disease, and conversely, distinct diseases may share common pathogenic mechanisms. Such novel revelations underscore the imperative of patient‐centric trial designs. The emerging paradigm in clinical trials should facilitate the real‐time alignment of optimal therapies with individual patient characteristics. Simultaneously, it should incorporate adaptive trial strategies that can flexibly adapt to the evolving disease conditions or newly discovered mechanistic insights, ensuring a more precise and effective approach to medical research and treatment.

### 1.2 Limitations of Traditional Clinical Trial Designs

The emergence of this innovative clinical trial paradigm challenges traditional approaches significantly, mainly due to three key limitations. First, traditional trials’ long timelines impede drug development, which lags behind the accelerating demand for medical and health innovations. The slow‐moving drug development process in traditional trials fails to meet the rapidly evolving needs of the medical field, delaying the availability of new treatments. Second, Phase I and II trials usually have small sample sizes. This often fails to represent the full diversity and complexity of patient populations, leading to potential selection bias. Inadequate sample sizes may cause inaccurate evaluations of drug efficacy and safety, as they cannot capture the responses of all relevant patient groups. Third, traditional trial designs are inflexible after initiation. Mid‐course adjustments are difficult, and the sequential independence of trial phases prevents integrating long‐term early‐stage outcomes into later studies. This lack of adaptability means that new information during the trial cannot be effectively utilized. Collectively, these challenges underscore the urgent need for transformative changes in clinical trial methodologies. Such changes are essential to meet the dynamic requirements of precision medicine, ensuring personalized and effective treatments.

### 1.3 The Connotation of Re‐Innovation in Clinical Trials

In this article, through analysis of innovative clinical trial cases, we propose the concept of “re‐innovation in clinical trials”, which encompasses two key dimensions: Deep integration of precision medicine's understanding of disease mechanisms; Structural innovation in trial paradigms. The emergence of innovative clinical trial designs, such as basket trials, umbrella trials, platform trials, and first‐in‐human multiple expansion cohorts, has revolutionized the evaluation and development of treatments. These designs offer numerous advantages, including targeted treatment evaluation, personalized medicine strategies, enhanced methodological flexibility, and accelerated drug development timelines. Their potential to transform clinical research practices and improve patient outcomes is immense.

### 1.4 Factors Driving the Development of Re‐Innovation Clinical Trials

The development of innovative clinical trials is mainly propelled by three interrelated factors. First, biomarker‐based technologies are constantly advancing. This progress allows for accurate molecular profiling of patients, providing a more precise basis for treatment. Second, an exploration of multi‐arm multi‐stage (MAMS) trial models is conducted. These models can concurrently assess multiple treatment strategies and adapt dynamically to real‐time data, optimizing the evaluation of treatments. Third, great emphasis is placed on improving trial flexibility and adaptability. This addresses patient heterogeneity and incorporates emerging evidence, ensuring the trials better meet the diverse needs of patients. These factors are molding the future of clinical trials and facilitating more effective and personalized healthcare.

## 2 Biomarker‐Based Precision Medicine: Molecular‐Feature Diagnosis and Treatment

### 2.1 Classification and Functions of Biomarkers

Biomarkers are objectively measurable and evaluable indicators that reflect physiological or pathological processes, as well as biological responses to exposure or therapeutic interventions. According to their functions, biomarkers can be primarily categorized into diagnostic, prognostic, predictive, and safety biomarkers. Based on their molecular types, biomarkers can be classified into protein, metabolite, nucleic acid, and lipid biomarkers. These molecular categories correspond to popular detection techniques in recent years, such as proteomics, metabolomics, genomics, and transcriptomics. Molecular‐feature diagnosis and treatment, particularly through next‐generation sequencing technology, represent a transformative shift in clinical diagnosis, treatment, and trial design [1, 2]. By analyzing gene sequences, epigenetic regulation mechanisms, posttranscriptional protein modifications, and multi‐protein interactions, researchers can construct individualized molecular profiles for patients. This approach not only enables novel diagnostic methods, prognostic assessments, and treatment targets but also challenges traditional disease classifications by uncovering molecular connections between seemingly distinct diseases.

### 2.2 Biomarker‐Driven Clinical Trials: Cases and Challenges

Molecular pathological typing has been widely applied in the precision diagnosis and treatment of tumors, and is actively reshaping the design paradigm of clinical trials. A demonstration of this paradigm is the BFAST trial, a global Phase II/III study integrating liquid biopsy for patient selection in advanced non‐small cell lung cancer (NSCLC) [3, 4, 5]. By prospectively screening 5220 patients via circulating tumor DNA (ctDNA) analysis, investigators identified 92 ROS1‐positive NSCLC cases (1.8% prevalence) who received entrectinib therapy. This trial design allowed precise targeting of ROS1 fusion‐positive tumors, achieving an 81.5% confirmed objective response rate and 14.8‐month median progression‐free survival (PFS) in the independent review. The BFAST trial has confirmed that ctDNA (liquid biopsy techniques) can be comparable to tissue biopsy techniques, making precision treatment for patients no longer completely dependent on obtaining lesion tissues. This precision medicine has the potential to redefine disease networks and classification criteria, ultimately enhancing therapeutic precision.

Liquid biopsy is not only applied in diagnostic and therapeutic stratification, but also helps monitor tumor response. A phase I clinical trial used histone H3.3K27M (H3K27M) mutation as a key biomarker, dynamically tracking the variant allele fraction (VAF) of H3.3K27M through serial collection of cf‐tDNA in cerebrospinal fluid (CSF) and plasma, detected by digital droplet polymerase chain reaction. The trial specifically set up arms to compare cf‐tDNA of H3K27M mutation in CSF (sensitivity 96.5%) and plasma (sensitivity 85.4%) for dual molecular validation. Longitudinal comparison between tumor area measured by MRI (using modified RANO criteria) and cf‐tDNA VAF showed that VAF “spikes” (increase ≥ 25%) predicted tumor progression 1‐3 months in advance (50% cases in plasma, 45.4% in CSF), and distinguished pseudoprogression from true response. Through the design framework of “dual sample types + dynamic molecular markers + imaging correlation,” this trial deeply integrated liquid biopsy into clinical trials, not only validating the clinical value of H3K27M cf‐tDNA but also providing a replicable paradigm for biomarker‐driven precision trials [6].

The FUTURE series of clinical trials employs a “one‐drug‐one‐target” framework, testing different biomarker‐guided treatment regimens simultaneously within the same trial to enhance efficiency. Based on immunohistochemical and next‐generation sequencing results, this framework classifies triple‐negative breast cancer (TNBC) into four major subtypes: luminal androgen receptor (LAR), immune‐regulated, basal‐like immune‐suppressed, and mesenchymal‐like, further divided into five treatment cohorts according to gene mutations to synchronously evaluate the clinical value of biomarkers such as HER2, PI3K/AKT, and immune microenvironment. This classification system breaks through the single‐biomarker design of traditional clinical trials, covers the molecular heterogeneity of TNBC, and avoids the limitations of conventional single‐arm trials. The study also uses ctDNA for adjuvant prognostic research, showing that gene mutations such as NF1, PIK3R1, ATR, and POLE are associated with poor prognosis. Results indicate that compared with traditional chemotherapy, biomarker‐based subtype treatment extends the median PFS from 5.8 months to 11.3 months (HR = 0.44, *p* < 0.0001), with benefits observed in all subtypes. For example, LAR‐PI3K/AKT mutant patients treated with everolimus combination therapy achieved a median PFS of 13.9 months, superior to 6.1 months with chemotherapy alone. Based on FUTURE‐SUPER results, three phase II/III trials (NCT05760378, NCT05806047, NCT05954442) have been initiated, targeting immune‐regulated, basal‐like immune‐suppressed, and LAR subtypes of TNBC to validate the long‐term benefits of biomarker‐guided therapy. Through multi‐dimensional biomarker integration, subtype‐specific treatment design, and dynamic monitoring systems, the FUTURE‐SUPER trial has established a clinical trial paradigm for precision treatment of TNBC, providing a replicable methodology for biomarker‐driven individualized cancer therapy. Its core value lies in upgrading biomarkers from diagnostic tools to the core basis for treatment decisions, accelerating target validation through trial design, and promoting the transformation of TNBC treatment from “empirical medicine” to “precision medicine” [7].

Despite its promise, biomarker‐driven clinical trials face significant challenges. First, during trial design, rigorous consideration must be given to biomarker selection methodologies while proactively addressing potential inaccuracies in measurement processes—particularly the impact of false positive rates—to prevent biased outcomes [8]. In studies involving complex patient populations or multiple disease types, ensuring comparability of biomarker assay accuracy across different disease categories becomes critical [9]. Furthermore, the front‐end process of biological specimen collection must adhere to principles of simplicity and feasibility. Innovative trial designs often involve expanded sample types, larger volumes, and more complex disease spectra. Only by ensuring stable and consistent specimen collection protocols can subsequent biomarker analysis achieve reliable results (Table 1).

**Table 1:** The table presents the definitions, advantages, and examples of four common innovative clinical trial designs.

Type	Arm design	Endpoint determination	Complexity management	Examples	References
Basket trial	For a single treatment, different diseases correspond to the same biomarker, with trial arms divided according to disease type	According to the natural history of each disease and standardized indicators such as remission rate, survival rate, and reduction rate.	Standardized biomarker testing; integration of multi‐disease data; ethics and regulation across disease groups	1. KY100001K (IDH inhibitor, cholangiocarcinoma, glioma, etc.)	[10, 11, 12]
				2. HSK42360 (BRAF V600E inhibitor, recurrent glioma/pancreatic cancer)	
Umbrella trial	For a single disease, different biomarkers correspond to different treatments, with grouping based on biomarkers	Based on unified endpoints for the same disease, such as OS and PFS, appropriate additional indicators for each arm are separately evaluated, such as cytokines and cell subtypes.	Standardized biomarker testing; Monitoring adverse events in different groups; Approval based on target‐therapy rationale	1. plasmaMATCH (advanced breast cancer, ctDNA‐based stratification)	[3, 4, 5, 13]
				2. BFAST (NSCLC, ctDNA‐based stratification)	
Platform trial	Multiple preset arms and a unified control group allow for adding, deleting, or modifying experimental arms, grouped according to biomarkers and disease classifications.	Including universal core indicators applicable to all arms and specific indicators reflecting the uniqueness of the treatment.	Adaptive statistical model; multi‐party multi‐center supervision; basis for protocol modification	ESMART (pediatric precision oncology, Bayesian‐based dose adjustments)	[14, 15, 16, 17, 18]

## 3 Multi‐Arm and Multi‐Stage Designs: Shared Targets and Pathways

### 3.1 Introduction to Common Innovative Clinical Trial Designs

Innovative trial designs, such as basket trials, umbrella trials, and platform trials, aim to overcome the limitations of traditional approaches by evaluating multiple treatment regimens concurrently. The MAMS design is central to this innovation, enabling the study of dynamically changing treatment arms within a single trial framework. Treatment arms that fail to demonstrate efficacy can be terminated early, while promising regimens are advanced.

### 3.2 Cases and Challenges of Multi‐Arm and Multi‐Stage Designs

The essence of multi‐arms lies in shared molecular targets and signaling pathways. By identifying core disease drivers and dissecting their roles across diverse physiological and pathological contexts, researchers can develop pan‐disease therapies. Multi‐stage designs, on the other hand, inherently involve adaptive trial management, including real‐time optimization of sample allocation, dose adjustments, and even addition of new arms based on emerging data.

Compared with traditional trial designs, the MAMS design adopted in the European AcSé‐ESMART trial [14, 19] has demonstrated significant advantages. By operating 16 treatment arms (such as Arm A to Arm Q) in parallel, the trial efficiency has been significantly improved. The treatment arms share a unified technical specification and legal framework. From 2016 to 2024, 255 patients from 6 countries were enrolled. The dose exploration and activity evaluation of different targeted drug combinations, such as CDK4/6 inhibitor combined with chemotherapy (Arm A) and WEE1 inhibitor combined with carboplatin (Arm C), were integrated into the same trial system, avoiding repeated construction, making efficient use of clinical resources such as molecular profiling platforms, and greatly shortening the clinical validation cycle of new drugs. Aiming at the complex biological characteristics of pediatric tumors, in which most of them have multiple variants with unclear targets and only 5% of recurrent tumors have clear driver mutations, the parallel multi‐arm can simultaneously explore the targeting strategies of different molecular pathways such as cell cycle and DNA repair, and the multi‐stage design allows dynamic adjustment of the protocol according to the trial data. Through the molecular enrichment strategy, patients with specific gene variations were included in each arm, and the sequencing data of responding patients were retrospectively analyzed to systematically explore potential predictive biomarkers. The integration of multi‐arm data can also identify cross‐pathway collaborative targets, promoting the transformation from single‐target to combination therapy. In view of the characteristics of the small number of pediatric oncology patients, the ESMART trial shared patient pools to expand the enrollment range, and dynamically adjusted the dose through the Bayesian adaptive design to balance innovation and safety. The trial established a unified clinical research network to achieve standardized management of sites in six countries, unified efficacy evaluation criteria and molecular profiling processes, accelerated cross‐border approval, established international standards for pediatric oncology research, and has been referenced or incorporated by many countries. This international perspective and global management approach are worthy of reference and emulation.

Interim analysis represents a core component of MAMS design, balancing scientific rigor and practical feasibility through “stage‐specific data‐driven decision‐making.” In essence, it employs statistical methods to dynamically screen effective arms and terminate ineffective ones during trial progression. The necessity of this strategy stems from the inherent contradiction between power (*the statistical ability to detect true efficacy*) and sample size (*trial costs*)—while larger sample sizes enhance the probability of identifying true efficacy, they significantly increase resource consumption. Choodari‐Oskooei et al. [17] systematically elaborated on the mathematical foundations and calculation methods of interim analysis in their research. Interim analysis timepoints are typically determined based on “information time,” benchmarked by the completion proportion of the control group sample size. For example, the phase II ROSSINI‐2 trial (NCT03838575) initiated its first interim analysis upon completing 21% of the control group sample and triggered the second analysis at 45% completion. As the control group in multi‐arm trials serves as the comparative benchmark for all study arms, using the control group's sample completion proportion as the trigger condition ensures that each study arm is evaluated under the same “information accumulation level,” avoiding timing biases caused by differences in recruitment rates across arms. Interim analyses at different stages correspond to specific stage‐wise significance levels (*e.g., α* = *0.4 for stage 1, α* = *0.14 for stage 2*), with significance levels decreasing monotonically to control the overall type I error rate. The Z‐test is adopted to calculate statistics, a parametric hypothesis testing method based on the standard normal distribution (*Z‐distribution*). It measures the degree of difference between sample statistics and population parameters by computing the test statistic Z (*Z*_*jk*_ =* θ*_*jk*_ */σ*_*jk*_), where ***θ***_***jk***_ represents the estimated efficacy difference between study arm *k* and the control group at stage *j* (e.g., infection rate difference, ratio of OS/PFS), and *σ*_*jk*_ denotes the standard error of this estimated difference. If the statistic *Z* exceeds the preset threshold, the arm is deemed “lacking benefit” and recruitment is halted. The futility boundary for stage 1 corresponds to α = 0.4 (*l*_*1*_ = *−0.253, derived from the normal distribution table allowing a 40% misjudgment rate*), indicating insufficient efficacy when the standardized value of the arm's effect size exceeds this threshold. This approach yielded tangible results in ROSSINI‐2: by screening at 21% and 45% milestones, the maximum sample size was reduced from the standard design's 8847 to 6613, while maintaining an overall power of 0.848 and a family‐wise error rate of 0.025—validating the scientific rationality of these timepoints. Upholding a power of 0.848 and a family‐wise error rate of 0.025 constitutes the “gold standard” of MAMS design: the former ensures the trial does not miss potential therapies (*84.8% probability of identifying true efficacy*), and the latter guarantees scientific reliability (*≤ 2.5% probability of misjudging an ineffective arm as beneficial*).

The ROSSINI‐2 trial also adopted an arm‐screening criterion to further control overall power and sample inclusion costs. The standard MAMS design for ROSSINI‐2 initially had a maximum sample size of 8847 cases, exceeding the budget. Through simulation of different screening rules, the 7:5:3 rule was found to effectively reduce sample size. This rule specifies that the trial starts with 7 study arms (*1 control arm* + *7 intervention arms*), requiring simultaneous evaluation of each arm's efficacy potential. After the first‐stage interim analysis, at most the top 5 arms by efficacy that do not cross the futility boundary (*α* = 0.4, *Z*_*j*_ < −0.253) are allowed to proceed. After the second‐stage interim analysis, at most the top 3 arms by efficacy that do not cross the futility boundary (*α* = *0.14, Z*_*j*_ < −*1.08*) are retained. This dynamic optimization mechanism enables MAMS designs to balance efficiency and scientific rigor in complex disease research, providing critical methodological support for clinical trials in the precision medicine era.

## 4 Flexibility and Adaptability in Trial Design

### 4.1 Real‐Time Monitoring System Ensures the Adaptability of the Trial

Establishing a real‐time monitoring system is of utmost importance during the conduct of clinical trials. It allows for dynamic monitoring of various patient indicators, including efficacy indicators (such as tumor size changes and improvement in biochemical markers), safety indicators (such as adverse reactions and abnormalities in hematological parameters), and biomarker alterations. Timely collection of feedback information enables researchers to promptly identify issues or emerging trends, such as a high incidence of adverse drug reactions or treatment outcomes that fall short of expectations. In response, the trial protocol can be promptly modified by adjusting drug dosages and treatment regimens, incorporating additional medications not originally planned, or optimizing the follow‐up protocol.

### 4.2 Management Requirements for Adaptive Trials

The ICH‐E8(R1) guideline underscores that definitions of clinical trial phases are intended to describe rather than prescribe, acknowledging that different stages of drug development may overlap or integrate. When new data emerge, additional studies may be warranted to ensure the integrity of the development process. ICH‐E9 outlines a framework for adaptive protocol optimization. This approach involves making pre‐defined adjustments to elements such as sample size, dosing regimens, or treatment arms, all within established boundaries. The goal is to avoid making frequent and disruptive changes to the master protocol, thereby maintaining the stability and reliability of the trial while allowing for necessary flexibility. When protocol modifications are indeed necessary, they must be accompanied by clear documentation of the scientific rationale in formal amendment documents. Simultaneously, it is essential to thoroughly assess the operational implications of these changes to minimize potential disruptions to the trial workflow.

Data management is another critical aspect. Both planned and unplanned monitoring scenarios require strict controls over access to unblinded data. These measures safeguard against unauthorized exposure, protecting the trial's integrity and participants' privacy. The FDA's Master Protocols Guidance requires that relevant trial documents be submitted via the electronic Common Technical Document system. This guidance also details key processes for protocol amendments, ethical review, informed consent updates, and safety reporting. Doing so ensures that clinical trials uphold scientific rigor while providing robust protection for study participants.

## 5 Expanded Application and Innovative Transformations

Sustained efforts in expanding applications and translational innovations represent a cornerstone of advancing modern medicine, exemplified by chlorogenic acid (CHA, 5‐O‐caffeoylquinic acid)—a polyphenol ubiquitously found in *Eucommia ulmoides* and coffee plants. CHA exerts therapeutic effects on multiple chronic conditions by regulating the AMPK‐Nrf2‐NFκB axis. In high‐fat diet mice, CHA activates AMPK, which leads to the downregulation of NFκB phosphorylation and hepatic inflammation [20]. In streptozotocin‐induced diabetic rats, CHA upregulates Nrf2 expression, thereby alleviating oxidative damage [21]. In Alzheimer's disease models, CHA‐mediated Nrf2 activation reduces Aβ‐induced NFκB activation and ameliorates neurodegeneration [22]. The compound also inhibits tumor progression through multi‐pathway synergistic effects including cell cycle arrest at G1/S phase via AMPK/mTOR signaling [23], apoptosis induction through mitochondrial pathway activation [24], ROS accumulation combined with antioxidant enzyme upregulation via Nrf2 [25], immunomodulatory effects promoting M1 macrophage polarization and T‐cell infiltration [26], and microglial phenotype modulation [27]. Supported by China's 13th Five‐Year National Science and Technology Major Project (2016ZX09101017), CHA is currently undergoing multi‐cancer clinical trials in NSCLC, hepatocellular carcinoma, prostate, colorectal, and gastric cancers. Notably, in glioblastoma patients, CHA treatment demonstrated a median OS of 11.3 months ‐ a significant improvement over standard therapy (5.7–7.5 months) [28]. An unexpected dermatological benefit was observed through CHA‐mediated regulation of collagen synthesis and metabolism, expanding its clinical utility [29, 30].

Honokiol is a natural compound derived from the bark of Magnolia officinalis. During clinical trials, it has exhibited a wide range of biological effects [31]. In Phase I and II clinical trials focusing on Late‐stage malignant solid tumors, especially gliomas (CTR20240113 and CTR20170822) [32], researchers made an unexpected observation: honokiol not only promoted hair growth in patients but also restored pigmentation to white hair. Further investigation revealed that the Wnt3a/β‐catenin signaling pathway plays a pivotal role in this phenomenon [33]. Thus, exploring the potential new indications of existing drugs is a crucial strategy for continuously enriching and advancing clinical treatment methodologies.

Focusing on patients and innovating the route of administration can enhance patient compliance and tolerance. In a Phase I clinical trial evaluating the oncolytic virus OH2 for the treatment of relapsed central nervous system tumors, the research team adopted an innovative approach by utilizing tumor cavity administration via the Ommaya reservoir [34]. Traditional administration methods for oncolytic viruses typically include intrathecal injections through lumbar puncture or direct delivery during surgical procedures [35]. Administering via the Ommaya reservoir not only significantly enhances patient tolerance but also facilitates the regular extraction of CSF, allowing dynamic monitoring of biomarkers. This approach is conducive to a deeper understanding of the drug's mechanism of action within the body, as well as the progression of the disease.

## 6 Discussion on Innovative Clinical Trials

In 2017, Bugano et al. [36] evaluated the impact of phase I trial expansion cohorts on phase II success and FDA approval, proposing the optimal sample size for expansion cohorts to provide specific numerical references for clinical design, which is highly practical. Focusing on the transformation efficiency from phase I to phase II, this study aligns with the industry demand for accelerating drug development, offering reference value for decision‐making in pharmaceutical companies. However, it does not explore the underlying reasons for expansion cohorts improving success rates, such as early toxicity screening and biomarker validation. In 2020, Park et al. [37] focused on the design principles, key cases, and regulatory approvals of basket and umbrella trials, emphasizing practical points like biomarker accuracy and sample size calculation, and providing application scenarios for statistical models (such as the Simon two‐stage design). In 2022, Fountzilas et al. [38] provided a comprehensive review of clinical trial designs in precision oncology, covering the full spectrum from traditional to next‐generation designs. Integrating numerous clinical cases (such as I‐SPY2), it merges molecular biology, statistics, and clinical practice, emphasizes the application of AI and real‐world data, and prospectively explores technology‐driven design innovations. It differentiates the clinical value of different trial designs, such as the advantages of platform trials in resource integration, and compares the efficacy of randomized and non‐randomized designs. Based on the integration of previous theories, we propose the concept of “re‐innovation of clinical trial designs.” Our research compiles classic cases from Asia and Europe and highlights noteworthy statistical issues. We argue that the core of innovative clinical trials lies in optimizing new drug research and development processes through MAMS trial designs to benefit patients, expanding new therapeutic prospects in ongoing research, and implementing them rapidly. Additionally, patient diagnosis and treatment are stratified through biomarkers to optimize inclusion and exclusion criteria, while study efficacy and error are controlled via sound statistical protocols. This constitutes our concept of “re‐innovation in clinical trial designs.”

## 7 Conclusion

The re‐innovation of clinical trial designs is driven by biomarker‐based precision medicine, MAMS designs, flexibility and adaptability, and expanded applications through innovative transformations. These elements are reshaping the future of clinical research, enabling more personalized and effective treatments. As these innovations continue to evolve, they hold the promise of improving patient outcomes and advancing the field of precision medicine.

## Author Contributions

**Shenglan Li:** conceptualization (equal), writing – original draft (equal), writing – review and editing (equal). **Jiachen Wang:** writing – original draft (equal). **Zhuang Kang:** conceptualization (equal), methodology (equal), project administration (equal). **Xun Kang:** conceptualization (equal), project administration (equal). **Feng Chen:** project administration (equal), resources (equal); supervision (equal). **Wenbin Li:** conceptualization (equal), project administration (equal), resources (equal), supervision (equal).

## Ethics Statement

The authors have nothing to report.

## Consent

The authors have nothing to report.

## Conflicts of Interest

The authors declare no conflicts of interest.

## Data Availability

Data sharing is not applicable to this article, as no data sets were generated or analyzed during the current study.

## References

[1] V. Subbiah and R. Kurzrock , “Universal Genomic Testing Needed to Win the War Against Cancer,” JAMA Oncology 2, no. 6 (2016): 719–720, 10.1001/jamaoncol.2016.0078. 27078832

[2] E. S. Lander , L. M. Linton , B. Birren , et al., “Initial Sequencing and Analysis of the Human Genome,” Nature 409, no. 6822 (2001): 860–921, 10.1038/35057062. 11237011

[3] S. Peters , R. Dziadziuszko , A. Morabito , et al., “Atezolizumab Versus Chemotherapy in Advanced Or Metastatic NSCLC With High Blood‐Based Tumor Mutational Burden: Primary Analysis of BFAST Cohort C Randomized Phase 3 Trial,” Nature Medicine 28, no. 9 (2022): 1831–1839, 10.1038/s41591-022-01933-w. PMC9499854 35995953

[4] S. Peters , S. M. Gadgeel , T. Mok , et al., “Entrectinib in ROS1‐Positive Advanced Non‐Small Cell Lung Cancer: The Phase 2/3 BFAST Trial,” Nature Medicine 30, no. 7 (2024): 1923–1932, 10.1038/s41591-024-03008-4. PMC11271410 38898120

[5] S. Y. Kim and R. S. Herbst , “BFAST but Be Smart: BTMB Remains an Exploratory Biomarker in NSCLC,” Nature Reviews Clinical Oncology 20, no. 1 (2023): 3–4, 10.1038/s41571-022-00698-y. 36271141

[6] E. Cantor , K. Wierzbicki , R. S. Tarapore , et al., “Serial H3K27M Cell‐Free Tumor DNA (Cf‐tDNA) Tracking Predicts ONC201 Treatment Response and Progression in Diffuse Midline Glioma,” Neuro‐Oncology 24, no. 8 (2022): 1366–1374, 10.1093/neuonc/noac030. 35137228 PMC9340643

[7] L. Fan , Z.‐H. Wang , L.‐X. Ma , et al., “Optimising First‐Line Subtyping‐Based Therapy in Triple‐Negative Breast Cancer (FUTURE‐SUPER): A Multi‐Cohort, Randomised, Phase 2 Trial,” Lancet Oncology 25, no. 2 (2024): 184–197, 10.1016/S1470-2045(23)00579-X. 38211606

[8] G. M. Clark , “Prognostic Factors Versus Predictive Factors: Examples From a Clinical Trial of Erlotinib,” Molecular Oncology 1, no. 4 (2008): 406–412, 10.1016/j.molonc.2007.12.001. 19383314 PMC5543832

[9] L. Bubendorf , S. Lantuejoul , A. J. de Langen , and E. Thunnissen , “Nonsmall Cell Lung Carcinoma: Diagnostic Difficulties in Small Biopsies and Cytological Specimens: Number 2 in the Series “Pathology for the Clinician” Edited by Peter Dorfmüller and Alberto Cavazza,” European Respiratory Review 26, no. 144 (2017): 170007, 10.1183/16000617.0007-2017. 28659503 PMC9488516

[10] K. Klas , K. Strzebonska , L. Zaborowska , et al., “Risk and Benefit for Basket Trials in Oncology: A Systematic Review and Meta‐Analysis,” Targeted Oncology 20, no. 1 (2025): 89–101, 10.1007/s11523-024-01107-3. 39455508 PMC11762573

[11] R. Ullah , Q. Yin , A. H. Snell , and L. Wan , “RAF‐MEK‐ERK Pathway in Cancer Evolution and Treatment,” Seminars in Cancer Biology 85 (2022): 123–154, 10.1016/j.semcancer.2021.05.010. 33992782

[12] X. Hu , W. Li , K. Zeng , et al., “Phase 1 Dose‐Escalation Study to Evaluate the Safety, Tolerability, Pharmacokinetics, and Anti‐Tumor Activity of FCN‐159 in Adults With Neurofibromatosis Type 1‐Related Unresectable Plexiform Neurofibromas,” BMC Medicine 21, no. 1 (2023): 230, 10.1186/s12916-023-02927-2. 37400844 PMC10318822

[13] N. C. Turner , B. Kingston , L. S. Kilburn , et al., “Circulating Tumour DNA Analysis to Direct Therapy in Advanced Breast Cancer (plasmaMATCH): A Multicentre, Multicohort, Phase 2a, Platform Trial,” Lancet Oncology 21, no. 10 (2020): 1296–1308, 10.1016/s1470-2045(20)30444-7. 32919527 PMC7599319

[14] B. Geoerger , F. Bautista , N. André , et al., “Precision Cancer Medicine Platform Trials: Concepts and Design of AcSé‐ESMART,” European Journal of Cancer 208 (2024): 114201, 10.1016/j.ejca.2024.114201. 39018630

[15] N. W. Clarke and N. D. James , “How to Compose Platform Trials,” European Urology Focus 9, no. 5 (2023): 715–718, 10.1016/j.euf.2023.10.016. 37925327

[16] N. M. Noor , S. B. Love , T. Isaacs , R. Kaplan , M. K. B. Parmar , and M. R. Sydes , “Uptake of the Multi‐Arm Multi‐Stage (MAMS) Adaptive Platform Approach: A Trial‐Registry Review of Late‐Phase Randomised Clinical Trials,” BMJ Open 12, no. 3 (2022): e055615, 10.1136/bmjopen-2021-055615. PMC8915371 35273052

[17] B. Choodari‐Oskooei , A. Blenkinsop , K. Handley , T. Pinkney , and M. K. B. Parmar , “Multi‐Arm Multi‐Stage (MAMS) Randomised Selection Designs: Impact of Treatment Selection Rules on the Operating Characteristics,” BMC Medical Research Methodology 24, no. 1 (2024): 124, 10.1186/s12874-024-02247-w. 38831421 PMC11145876

[18] T. Burnett , F. König , and T. Jaki , “Adding Experimental Treatment Arms to Multi‐Arm Multi‐Stage Platform Trials in Progress,” Statistics in Medicine 43, no. 18 (2024): 3447–3462, 10.1002/sim.10090. 38852991

[19] B. Geoerger , X. Paoletti , F. Bautista , et al., “AcSé‐ESMART, a European Precision Cancer Medicine Proof‐of‐Concept Platform Trial,” Nature Medicine 29, no. 12 (2023): 2985–2987, 10.1038/s41591-023-02580-5. 37857712

[20] S. H.v., V. K , D. Patel , and S. K , “Biomechanism of Chlorogenic Acid Complex Mediated Plasma Free Fatty Acid Metabolism in Rat Liver,” BMC Complementary and Alternative Medicine 16, no. 1 (2016): 274, 10.1186/s12906-016-1258-y. 27495925 PMC4974694

[21] K. Karthikesan , L. Pari , and V. Menon , “Combined Treatment of Tetrahydrocurcumin and Chlorogenic Acid Exerts Potential Antihyperglycemic Effect on Streptozotocin‐Nicotinamide‐Induced Diabetic Rats,” General Physiology and Biophysics 29, no. 1 (2010): 23–30, 10.4149/gpb_2010_01_23. 20371877

[22] H. Zhu , F. Shen , X. Wang , H. Qian , and Y. Liu , “Chlorogenic Acid Improves the Cognitive Deficits of Sleep‐Deprived Mice *via* Regulation of Immunity Function and Intestinal Flora,” Phytomedicine 123 (2024): 155194, 10.1016/j.phymed.2023.155194. 37995532

[23] X. Wang , J. Liu , Z. Xie , et al., “Chlorogenic Acid Inhibits Proliferation and Induces Apoptosis in A498 Human Kidney Cancer Cells *via* Inactivating PI3K/Akt/mTOR Signalling Pathway,” Journal of Pharmacy and Pharmacology 71, no. 7 (2019): 1100–1109, 10.1111/jphp.13095. 30989669

[24] S. Sadeghi Ekbatan , X.‐Q. Li , M. Ghorbani , B. Azadi , and S. Kubow , “Chlorogenic Acid and Its Microbial Metabolites Exert Anti‐Proliferative Effects, S‐Phase Cell‐Cycle Arrest and Apoptosis in Human Colon Cancer Caco‐2 Cells,” International Journal of Molecular Sciences 19, no. 3 (2018): 723, 10.3390/ijms19030723. 29510500 PMC5877584

[25] L. Wang , H. Du , and P. Chen , “Chlorogenic Acid Inhibits the Proliferation of Human Lung Cancer A549 Cell Lines by Targeting Annexin A2 In Vitro and In Vivo,” Biomedicine & Pharmacotherapy = Biomedecine & Pharmacotherapie 131 (2020): 110673, 10.1016/j.biopha.2020.110673. 32882585

[26] X. Li , S. Zhu , P. Yin , et al., “Combination Immunotherapy of Chlorogenic Acid Liposomes Modified With Sialic Acid and PD‐1 Blockers Effectively Enhances the Anti‐Tumor Immune Response and Therapeutic Effects,” Drug Delivery 28, no. 1 (2021): 1849–1860, 10.1080/10717544.2021.1971797. 34515617 PMC8439241

[27] J. Wang , S. Li , Y. Chen , et al., “ScRNA‐Seq Unveils the Functional Characteristics of Glioma‐Associated Macrophages and the Regulatory Effects of Chlorogenic Acid on the Immune Microenvironment: A Study Based on Mouse Models and Clinical Practice,” Frontiers in Immunology 15 (2025): 1494806, 10.3389/fimmu.2024.1494806. 39867913 PMC11757274

[28] Z. Kang , S. Li , X. Kang , et al., “Phase I Study of Chlorogenic Acid Injection for Recurrent High‐Grade Glioma With Long‐Term Follow‐Up,” Cancer Biology & Medicine 20, no. 6 (2023): 465–476, 10.20892/j.issn.2095-3941.2022.0762. 37366368 PMC10291982

[29] R. Zhang , H. Li , W. Zhang , et al., “Chlorogenic Acid/Carboxymethyl Chitosan Nanoparticle‐Assisted Biomultifunctional Hyaluronic Acid‐Based Hydrogel Scaffolds for Burn Skin Repair,” International Journal of Biological Macromolecules 275, no. Pt 1 (2024): 133528, 10.1016/j.ijbiomac.2024.133528. 38945346

[30] N. Xue , Y. Liu , J. Jin , M. Ji , and X. Chen , “Chlorogenic Acid Prevents UVA‐Induced Skin Photoaging Through Regulating Collagen Metabolism and Apoptosis in Human Dermal Fibroblasts,” International Journal of Molecular Sciences 23, no. 13 (2022): 6941, 10.3390/ijms23136941. 35805942 PMC9266774

[31] A. Rauf , A. Olatunde , M. Imran , et al., “Honokiol: A Review of Its Pharmacological Potential and Therapeutic Insights,” Phytomedicine 90 (2021): 153647, 10.1016/j.phymed.2021.153647. 34362632

[32] S. Li , J. Chen , Y. Fan , et al., “Liposomal Honokiol Induces ROS‐Mediated Apoptosis *via* Regulation of ERK/P38‐MAPK Signaling and Autophagic Inhibition in Human Medulloblastoma,” Signal Transduction and Targeted Therapy 7, no. 1 (2022): 49, 10.1038/s41392-021-00869-w. 35185151 PMC8858958

[33] S. Li , J. Chen , F. Chen , et al., “Liposomal Honokiol Promotes Hair Growth *via* Activating Wnt3a/β‐Catenin Signaling Pathway and Down Regulating TGF‐Β1 in C57BL/6N Mice,” Biomedicine & Pharmacotherapy = Biomedecine & Pharmacotherapie 141 (2021): 111793, 10.1016/j.biopha.2021.111793. 34098216

[34] Y. Zheng , X. Wang , Q. Ji , et al., “OH2 Oncolytic Virus: A Novel Approach to Glioblastoma Intervention Through Direct Targeting of Tumor Cells and Augmentation of Anti‐Tumor Immune Responses,” Cancer Letters 589 (2024): 216834, 10.1016/j.canlet.2024.216834. 38537773

[35] J. Gállego Pérez‐Larraya , M. Garcia‐Moure , S. Labiano , et al., “Oncolytic DNX‐2401 Virus for Pediatric Diffuse Intrinsic Pontine Glioma,” New England Journal of Medicine 386, no. 26 (2022): 2471–2481, 10.1056/nejmoa2202028. 35767439

[36] D. D. G. Bugano , K. Hess , D. L. F. Jardim , et al., “Use of Expansion Cohorts in Phase I Trials and Probability of Success in Phase II for 381 Anticancer Drugs,” Clinical Cancer Research 23, no. 15 (2017): 4020–4026, 10.1158/1078-0432.ccr-16-2354. 28377482 PMC5540786

[37] J. Park , G. Hsu , E. G. Siden , K. Thorlund , and E. J. Mills , “An Overview of Precision Oncology Basket and Umbrella Trials for Clinicians,” CA: A Cancer Journal for Clinicians 70, no. 2 (2020): 125–137, 10.3322/caac.21600. 32031692 PMC7187272

[38] E. Fountzilas , A. M. Tsimberidou , H. H. Vo , and R. Kurzrock , “Clinical Trial Design in the Era of Precision Medicine,” Genome Medicine 14, no. 1 (2022): 101, 10.1186/s13073-022-01102-1. 36045401 PMC9428375

